# Biological Properties and Bioactive Components of *Allium cepa* L.: Focus on Potential Benefits in the Treatment of Obesity and Related Comorbidities

**DOI:** 10.3390/molecules24010119

**Published:** 2018-12-30

**Authors:** Mariangela Marrelli, Valentina Amodeo, Giancarlo Statti, Filomena Conforti

**Affiliations:** Department of Pharmacy, Health and Nutritional Sciences, University of Calabria, I-87036 Rende (CS), Italy; valentina.amodeo@unical.it (V.A.); giancarlo.statti@unical.it (G.S.); filomena.conforti@unical.it (F.C.)

**Keywords:** *Allium*, bioactive compounds, diabetes, hyperlipidaemia, obesity, onion, organosulfur compounds, quercetin

## Abstract

Common onion (*Allium cepa* L.) is one of the oldest cultivated plants, utilized worldwide as both vegetable and flavouring. This species is known to contain sulphur amino acids together with many vitamins and minerals. A variety of secondary metabolites, including flavonoids, phytosterols and saponins, have also been identified. Despite the predominant use of this plant as food, a wide range of beneficial effects have also been proved. Different biological properties, such as antioxidant, antimicrobial and antidiabetic, have been reported. The aim of this review is to provide an overview of the studies concerning the beneficial effects of this species against obesity and its related comorbidities, such as hyperlipidaemia, hypertension and diabetes. Both in vitro and in vivo results about onion dietary supplementation have been taken into account. Furthermore, this review examines the possible role of onion bioactive components in modulating or preventing weight-gain or related diseases, as well as the possible mechanisms behind their activity.

## 1. Introduction

The search for new tools against obesity is one of the main challenges of pharmaceutical research in the last decades [1,2,3]. Despite a variety of studies focusing on the management of this disease, its global incidence is raising, resulting in enormous social costs [4,5]. Obesity consists of an excessive fat accumulation in adipose tissues.

Beside the weight gain, obesity is a complex public health issue, as this pathological condition is linked to an increased risk of other diseases, including type 2 diabetes, cardiovascular diseases and cancer [6,7,8]. Obesity constitutes a pre-requisite for the so-called “metabolic syndrome,” defined as a combination of risk factors, such as obesity, insulin resistance, dyslipidaemia and hypertension, that together are able to cause serious outcomes, such as type 2 diabetes mellitus and cardiovascular diseases [9]. Diabetes is one of the greatest health care problems, that has been calculated to affect 387 million people worldwide in 2014 and that could potentially reach pandemic levels by 2030. A number of studies have underlined the close relationship between obesity and diabetes: it has been estimated that about 90% of type 2 diabetes is related to excessive body weight. Because of this strong association, the term “diabesity” has been coined [10].

Despite the huge investments for the development of effective anti-obesity agents, only a few drugs have been approved for marketing. Furthermore, several of them have been withdrawn due to their side effects, such as dinitrophenol, sibutramine or rimonabant [11,12].

Orlistat, introduced in 1998, was the first really successful drug against obesity. This molecule is a semisynthetic derivative of the natural lipase inhibitor produced by *Streptomyces toxytricini* and it is a potent pancreatic and gastric lipase inhibitor, able to prevent dietary fat absorption by 30%. However, also this drug might not be well tolerated, since some side effects, such as diarrhoea, faecal incontinence, flatulence, bloating and dyspepsia, commonly occur [2,13].

Recently, four other new drugs have been introduced: lorcaserin and phentermine+topiramate ER, approved by FDA in 2012 and successively naltrexone SR/bupropion SR and liraglutide recently approved in both the USA and Europe. Nevertheless, also with these drugs some patients may experience different adverse effects [14].

A negative correlation between the consumption of fruits and vegetables and the incidence of several diseases has been confirmed by many epidemiological studies. Plants are an important source of health-promoting compounds, such as vitamins and flavonoids. Because of the recognition of the importance of edible plants also for human health and not only as food, there is a great interest toward plant-derived pharmaceuticals, dietary supplements and functional foods [15,16].

Alongside other new anti-obesity drugs that are currently under investigation [17], the potential role of plants and their metabolites has been taken into account in most recent studies as an alternative in the treatment of obesity, with the aim to find well-tolerated natural effective drugs. A variety of natural products that includes medicinal plant extracts or isolated pure compounds is currently on the market. Different plants contain bioactive components with anti-obesity effects on body metabolism and for this reason they have been investigated and reported to be useful in treatment of obesity, diabetes and other chronic diseases [18].

Different classes of phytochemicals have been proved to modulate obesity through various mechanisms of action, such as polyphenols [19,20], terpenes [21,22] and saponins [23].

The activity of many effective herbal medicines in the management of obesity has been reported [24,25,26,27,28,29]. 

*Allium cepa* L. is one of leading vegetable crops in the world and the phytochemical and biological characteristics of this species have been deeply investigated. The purpose of this review is to present a comprehensive overview of recent and relevant studies related to the beneficial effects of *A. cepa* L. against obesity and its related comorbidities, such as hyperlipidaemia, hypertension and diabetes. Both in vitro and in vivo effects of onion dietary supplementation are presented and discussed. Moreover, the potential anti-obesity activity of onion bioactive components and their possible mechanisms of action are reported.

## 2. *Allium cepa* L.: Botany, Distribution and Phytochemistry

The species *Allium cepa* L., commonly known as onion, has been for a long time considered a member of the Liliaceae family [30,31] but according to more recent taxonomic schemes the genus *Allium* belongs to the family Amaryllidaceae, subfamily Allioideae [32]. This genus is one of the largest monocot genera as it comprises about 850 species [33].

*A. cepa* is a biennial plant with adventitious and fibrous roots and 3–8, distichous, glaucous leaves. The bulb is made of concentric, enlarged fleshy leaf bases. The outer leaf base dries and becomes thin and variously coloured, forming the protective coat, while the inner leaf bases thicken when the bulb develops. The mature bulb can be globose, ovoid or elongate and its size varies depending on the cultivar [34].

*A. cepa* is a bulbous plant widely cultivated in almost every country of the world. According to the last available FAO statistics [35], in 2016 the top producers of onions were China (23,849,053 tonnes) and India (19,415,425 t), followed by Egypt and USA (about 3,000,000 t), Iran, Turkey, Russian Federation, Pakistan, Bangladesh and Brazil (from 2,345,768 to 1,657,441 t). Onions produced in European countries accounted for 10.9% of the world production, being Asia (65.5%) the most important producer.

Because of its worldwide distribution, a great number of cultivars can be identified: ‘Stardust,’ ‘Snowpack,’ ‘Redlight,’ ‘Hytech’ [36], ‘Tropea,’ ‘Montoro’ [37], ‘Festival,’ ‘Castillo’ [38], ‘Nazik Red’ and ‘Ailsa Craig’ [39] are just some examples.

At present, approximately 13,000 onion accessions are held in gene banks worldwide. A high genetic variability can be observed regarding the morphological features [36].

Onion bulb is used as both vegetable and flavouring. The bulb is eaten raw or cooked. The leaves and the flowers of the plant are also edible and are often used in salads. The seeds of the plant are sprouted and eaten as well [34]. Onion is used as food and spice in diets of almost all cultural areas. In addition, onions can be processed into various products such as dehydrated powder or juice, which are used for the seasoning and flavouring of food [36].

Onion is rich in several phytonutrients that are recognized as important elements of the Mediterranean diet but it has received attention also for its biological properties and potential application in the treatment and prevention of a number of diseases [34]. *A. cepa* is known to contain many vitamins and minerals and is rich in sulphur amino acids. Moreover, a variety of secondary metabolites has been identified in this species, such as flavonoids (particularly flavonols and anthocyanin), phytosterols and saponins [40]. Many studies focused on the phytochemical composition of onions, which have been already extensively reviewed [41,42,43].

## 3. *A. cepa* Biological Activities

A variety of biological properties has been reported for *A. cepa*. A great number of studies focus on its antimicrobial potential, which has been already reviewed in dept by Teshika and colleagues [42]. However also other properties, such as antioxidant, anti-melanogenesis, antispasmodic and antiproliferative activities, have been attributed to this species.

The antioxidant properties of *A. cepa* have been deeply investigated and documented [44,45]. These studies are very important as recent findings have demonstrated a link between increased body weight and fat deposition and enhanced oxidative stress. The lower levels of antioxidant might play a role in the development of obesity comorbidities. It has been hypothesized that obesity could be inversely associated with antioxidant intake [46]. Moreover, antioxidants may be important in the management of the different obesity related diseases such as cardiovascular diseases and diabetes [47] Benkeblia [48] reported the radical scavenging and antioxidant properties of the methanolic extracts of different onion cultivars from Algeria: *A. cepa* ‘Premier’ (green onion), ‘Jaune d’Espagne’ (yellow onion), ‘Amposta’ (red) and ‘Rouge’ (purple).

The radical scavenging and antioxidant activities of extracts from skin and edible part of red onion *A. cepa* var. Red baron from Slovenia have been instead investigated by Škerget and colleagues [49]. The best radical scavenging potential was observed for the onion skin pure acetone extract, while the highest antioxidant activity, measured through the β-carotene bleaching test, was demonstrated for the onion skin extracted with 35% and 60% acetone and 60% ethanol. On the other hand, a low antioxidant activity was observed for onion edible part extracts in these experiments.

Santas and colleagues reported the antioxidant properties of three different Spanish *A. cepa* cultivars: white skinned onion ‘Fuentes de Ebro,’ white skinned onion ‘Calçot de Valls’ and yellow skinned onion ‘Grano de Oro’ [50]. Plant materials were extracted with 75% methanol and obtained extracts were then fractionated. Ethyl acetate subfractions contained the highest amount of flavonoids and the Trolox equivalent antioxidant capacity was reported to be 74.86, 24.59 and 4.55 µmol Trolox/g for ‘Grano de Oro,’ ‘Fuentes de Ebro’ and ‘Calçot de Valls,’ respectively.

A strong antioxidant activity was demonstrated for red onion ‘N-53′ from India [51]. A good antioxidant potential was also reported for the bulb methanolic extracts of three *A. cepa* cultivars: ‘Pusa Red’ (red), ‘Pusa White Round’ (white) and ‘Arka Pitamber’ (yellow) [52].

Also Benmalek and co-workers assessed the radical scavenging of *A. cepa*. An IC_50_ value equal to 2.91 × 10^−5^ mg/mL was reported for the outer layer of red onions [53].

The oxygen radical absorbance capacity of *A. cepa* has been also reported [54]. Pulp and skin were extracted with distilled water and 95% ethyl alcohol. The highest ORAC value and total phenolic content were detected for the ethyl alcohol extract of onion skin.

Lee and colleagues [55] evaluated the antioxidant properties of four different extracts from *A. cepa* peels: plant material was extracted with hot ethanol (60°C), hot water (80°C) and by means of subcritical water extraction at 110°C and 165°C. The ethanolic onion peel extract showed a better DPPH radical scavenging activity and a highest antioxidant activities determined by ferric thiocyanate assay compared to the other samples.

Also *A. cepa* essential oil showed antioxidant properties [56]. The essential oil was extracted by supercritical CO_2_ extraction and biological properties were assayed by means of ABTS assay (IC_50_ = 0.67 mg/mL), DPPH test (IC_50_ = 0.63 mg/mL) and metal chelating assay (IC_50_ = 0.51 mg/mL).

Interestingly, the potential activity of *A. cepa* against oxidative stress was verified also in vivo. Campos and co-workers [57] investigated the effects of the consumption of onion extract in STZ-induced diabetic rats. Onion bulbs were collected from Brazil and extracted with water by infusion (40 g/100 mL). It was demonstrated that onion intake was able to decrease superoxide dismutase activity while no increased lipid hydroperoxide and lipoperoxide concentrations were observed in treated diabetic rats.

The antioxidant potential of the ethanolic extract and fractions of *A. cepa* were studied by Baragob and co-workers both in vitro and in vivo [58]. In vitro activities were verified using DPPH and NO radical scavenging methods, whereas the in vivo effects on antioxidant enzyme were assessed in the erythrocytes and pancreas of normal and STZ-induced diabetic rats. Before treatment, normal groups contained higher enzyme levels of SOD, CAT, GSH and LPO than diabetic groups, while the administration of *A. cepa* ethanolic extract and its chloroform fraction in diabetic groups significantly increased the level of SOD, CAT and GSH and decreased LPO level to near normal erythrocytes and pancreas.

Ren and colleagues [59] assessed the in vitro antioxidant activity of two onion varieties (‘Hyskin’ and ‘Red Baron’) grown in a 6-year field study. The influence of conventional, organic and mixed cultivation practices on phytochemical composition and antioxidant activity was verified as well. Both varieties showed the best effectiveness under fully organic management.

Different onion by-products have also been reported to possess good antioxidant properties that make onion useful as functional food ingredient [60].

Moreover, Gawlik-Dziki and colleagues [61] investigated the antioxidant potential of breads enriched with *A. cepa* skin. The food supplement was prepared by drying onions (*A. cepa* ‘Wolska’) in an oven at 50 °C and by powdering the plant material using a laboratory mill. For the experiments, the flour used in the formula of control bread (wheat bread flour 600 g, type 750) was replaced with onion skin at 1%, 2%, 3%, 4%, 5% levels. Bioaccessibility and bioavailability were determined in vitro by using the human gastrointestinal tract model. Breads were then extracted with 80% methanol and bread extracts were assayed for their antiradical activity, their ability to inhibit lipid peroxidation, the metal chelating activity and the ferric reducing power. The antioxidant potential of onion enriched bread was significantly higher than that observed in the control.

Furthermore, Helen and co-workers [62] demonstrated that onion oil is an effective antioxidant against the oxidative damage caused by nicotine in rats to a similar extent to vitamin E.

The appropriate processing technologies and storage conditions able to preserve the antioxidant capacity of onions have been also investigated. Siddiq and co-workers [63] reported that the use of mild-heat treatment for processing fresh-cut onions (50 and 60 °C) did not affect the antioxidant activity (evaluated by means of ABTS and DPPH tests) and the colour of fresh-cut onions. Moreover, a good antioxidant activity has been recently demonstrated for onion polysaccharide fractions [64]

Anti-melanogenesis properties have been also reported for *A. cepa*. A strong melanin biosynthesis inhibitory activity on B16 melanoma cells was reported for the methanolic extract of the dried skin of *A. cepa* from Indonesia [65]. Quercetin and quercetin 4′-*O*-β-glucoside were the most active identified compounds. Obtained results demonstrated that *A. cepa* skin extract could likely act as a potent skin-whitening agent by inhibiting the melanin formation in B16 melanoma cells, similarly to arbutin, which was used as a positive control.

Interestingly, the fresh juice of onion bulbs was tested for its androgenic effects on the spermatogenesis cycle in Wistar albino rats [66]. Administration of 0.5 g/rat and 1 g/rat of freshly prepared onion juice significantly increased sperm motility and viability as compared to the control after 20 days of treatment. Serum total testosterone level was also increased.

Moreover, Naseri and co-workers investigated the antispasmodic activity of onion peel extract [67]. Onion peel powder was extracted with 70% ethanol trough maceration and the antispasmodic effects were evaluated on Wistar rat ileum contractility. Onion peel extract was able to reduce the KCl and carbachol-induced ileum contractions in a dose dependent manner.

Another potential biological property of *A. cepa* was investigated by Sakakibara and co-workers [68], who verified the antidepressant-like effect in a rat behavioural model of depression. Onions from Japan were peeled and processed to a powder with a freeze dryer. Rats were orally administered with *A. cepa* powder dissolved in water or with the synthetic antidepressant imipramine for 14 days of treatment. The immobility time in FST was significantly reduced by the administration of onion at a dosage of 50 mg/kg, similarly to imipramine.

*A. cepa* extract exerted also antiproliferative activity. Obesity has been associated with an increased risk of developing some forms of cancer, such as breast [69], endometrial [70], prostate [71] and colorectal cancer [72]. This relationship may be due to different mechanisms, depending on the cancer site, such as insulin resistance and alterations in circulating levels of steroid hormones [73].

Effectiveness on the human breast cancer MDA-MB-231 cells was reported by Wang and colleagues [74]. The ethyl acetate fraction of onion showed the best inhibition of cell viability, with an IC_50_ value equal to 52 μg/mL. This fraction, interestingly, was the most effective in inhibiting FAS in vitro, with an IC_50_ value equal to 2.4 ± 0.3 μg/mL. Western blotting analysis demonstrated that FAS was expressed in high level in MDA-MB-231 cells. The treatment with this fraction at the concentrations of 25 μg/mL and 50 μg/mL for 24 h induced a decrease of the intracellular FAS activities of MDA-MB-231 cells (56.3% and 32.1%) compared to the control. Obtained results seem to suggest that the apoptosis induced by onion ethyl acetate fraction might occur via inhibition of FAS.

The antiproliferative activity of *A. cepa* on MDA-MB-231 cells was also assessed by Fredotović and co-workers, who verified the antiproliferative effects of the methanolic extract of the plant on both these breast cancer cells and the human glioblastoma cell line-A1235A [75].

Moreover, *A. cepa* was also proved to be effective against the murine melanoma cell line B16F10 [76] and the human colorectal adenocarcinoma Caco-2 cell line [77]. The effects on human hepatocellular carcinoma cells HepG2 were tested as well [77,78].

## 4. Beneficial Effects of Onion in the Treatment of Obesity

The aim of this review is to provide an extensive overview of the studies concerning the potential anti-obesity activity of *A. cepa* extracts and their phytochemical constituents on obesity and related comorbidities. A summary of all the studies concerning the potential beneficial effects of this species is provided in Table 1.

### 4.1. Pancreatic Lipase Inhibition

The potential effects of *A. cepa* on pancreatic lipase were first taken into account by Kim and colleagues [79], who tested the skin extract of the plant. Alcoholic extract from dry powdered onion skin was spray-dried and dissolved in dimethylsulphoxide. Sample inhibited pancreatic lipase with an IC_50_ value of 53.70 mg/mL.

Slanc and co-workers [80] investigated instead the potential inhibitory activity of the hydroalcoholic extract from the leaves of this species. Sample was tested in vitro for its ability to inhibit porcine pancreatic lipase using both *p*-nitrophenylpalmitate and 5-bromo-4-chloro-3-indoxylpalmitate as substrates. However, an inhibition lower than 40% was reported.

More recently, the juice of onion was also tested for its lipase inhibitory potential. Onions were cut into small pieces, blended with water and centrifuged [81]. Sample was demonstrated to inhibit pancreatic lipase with an IC_50_ value equal to 9.5 mg/mL

### 4.2. Adipogenesis Inhibition

Yoshinari and co-workers investigated the effects of *A. cepa* on adipogenesis [82]. Analysed extract was obtained by heating fresh onion and then concentrating at 92 °C after squeezing. Sample was tested for its ability to inhibit the differentiation of rat white preadipocyte cells. Cells were incubated in medium containing insulin and different concentrations of *A. cepa* for seven days. Both extract and its sulphur-containing compounds showed an inhibitory activity, suggesting that analysed samples can suppress lipid accumulation or differentiation in adipocyte.

Moon and colleagues [83] demonstrated that quercetin-rich onion peel extract is able to suppress preadipocyte differentiation and to inhibit adipogenesis. To perform the experiments, onions from Korea were extracted with 60% aqueous ethanol solution. Concentrated extract was finally processed to a powder with a freeze dryer. Sample was able to decrease lipid accumulation in preadipocytes 3T3-L1 cells and to inhibit the differentiation of 3T3-L1 into adipocytes in a dose dependent manner.

The ability to suppress adipogenesis of quercetin-rich onion peel extract was further investigated by Bae and colleagues [84]. Hydroalcoholic extract was concentrated and processed with a spray dryer to produce a powder containing 150 mg quercetin/g. Experiments were performed using 3T3-L1 preadipocyte cells. It was demonstrated that onion peel extract and quercetin treatment significantly decreased lipid accumulation, as a lower level of intracellular lipid content was observed with treatment with peel extract compared with both the control and quercetin. Moreover, the treatment with onion peel extract significantly inhibited the activity of GPDH, an important enzyme involved in fatty acid and triacylglycerol synthesis in adipocytes, which increases during adipogenesis. Authors also reported that onion peel extract could suppress adipogenesis by down-regulating PPAR-γ and C/EBPα, some of the effectors that activate the process of adipogenesis.

### 4.3. Increase of Energy Expenditure

The increase in energy expenditure in response to caloric excess is called diet-induced thermogenesis. This important homeostatic mechanism restricts weight gain in response to caloric excess contributing to the relative stability of body weight [95,96].

Brown and beige adipose tissues are able to metabolize the stored chemical energy as heat. This process is named adaptive thermogenesis and is now considered a new potential target against obesity and associated metabolic disorders [97].

Lee and co-workers [85] reported that onion is able to modify the characteristics of white adipocytes to those of brown-like adipocytes both in vivo, in the white adipose tissue of C57BL/6 mice and in vitro, in 3T3-L1 fibroblasts. Dried onion peels were pulverized and extracted with 60% aqueous ethanol in an ultrasonic bath. The retroperitoneal and subcutaneous adipose tissues of 0.5% onion-peel-extract-fed mice showed an increased expression of brown adipose tissue-specific genes. The same effect was induced in vitro in 3T3-L1 adipocytes.

## 5. Effects on Obesity Related Comorbidities

Obesity has been associated with a decreased life expectancy as it is related to an increased incidence of other pathological conditions, such as dyslipidaemia, cardiovascular disorders, type II diabetes and cancer [7,8,98,99].

Obesity constitutes a pre-requisite for the so-called “metabolic syndrome.” Actually, it has been observed a prevalence of this syndrome in obese and overweight individuals. It is characterized by multiples biochemical, clinical and metabolic factors, such as dyslipidaemia, hypertension, glucose intolerance and proinflammatory state, which directly increase the risk of atherosclerotic cardiovascular diseases and type 2 diabetes mellitus, thus causing mortality [9,100].

For these reasons, the beneficial effects of *A. cepa* on these pathological conditions have also been taken into account in the present review. A number of studies focus on the potential effects of this plant species in the treatment of more than one of these diseases.

For example, it is interesting to note that Kim and co-workers [86] verified the effects of onion peel extract consumption on the expression of inflammatory mediators from the adipose tissue in high fat diet induced obese animal model. Utilized onions were from Korea and were extracted with hydroalcoholic solution (ethanol 60%). Obtained solution was concentrated and processed with a freeze dryer, to obtain a powder containing 100 mg quercetin/g. Dawley rats were divided into three groups: control, HF and high fat diet with onion peel extract group. After 8 weeks of treatment, perirenal and epididymal fat weights were not significantly affected, while the weight of mesenteric fat was significantly lower in the group fed with high fat diet and onion peel extract compared with the HF group. In order to assess if onion peel extract consumption could have beneficial effects on obesity-induced inflammation, changes in adipokine mRNA (adiponectin, IL-6 and visfatin) and PPAR-γ2 levels from adipose tissues were measured. Obtained results showed an influence of adipokine expressions, particularly from mesenteric fat. A higher adiponectin mRNA levels were detected in the group fed with high fat diet and onion supplementation compared with the other groups. Moreover, in animals that received onion peel extract supplementation, the levels of IL-6 mRNA levels were slightly lower than those in the HF group. This study assessed the potential beneficial effects of *A. cepa* on the expression of inflammatory mediators from the adipose tissue. In fact, the adipose tissue is not only important for the energy storage but also plays a role in regulating physiologic and pathologic processes, such as inflammation. This tissue releases many proinflammatory molecules implicated in the development of obesity comorbidities including insulin resistance and the increased risk of cardiovascular diseases [101]. 

### 5.1. Hyperlipidaemia

The potential health benefits of *A. cepa* on hyperlipidaemia were taken into account already at the beginning of the nineties [87], when Lata and co-workers demonstrated that the oral administration of the onion petroleum extract in albino rats significantly prevented the increased serum cholesterol and serum triglyceride levels caused by an atherogenic diet.

The antihyperlipidemic activity of *A. cepa* was also reported by Kim and co-workers [79]. The authors investigated the potential health benefits of *A. cepa* in vivo on mice. *A. cepa* skin extract effectively decreased plasma triacylglycerol level elevation after administration of a lipid emulsion in rats and it was also able to prevent the body weight gain induced by a high-fat diet.

The effects of *A. cepa* on lipid metabolism were also assessed by Lee and co-workers [88]. Male SD rats fed a high fat and high cholesterol diet were treated with an onion powder supplementation. The amount of total cholesterol in the liver of treated rats decreased significantly in the high fat and onion powder intake fed group compared to group fed with a high fat diet (80.9 ± 7.0 and 90.3 ± 3.9 mg/g wet wt, respectively).

Baragob and co-workers [58] investigated the antihyperlipidemic potential of the ethanolic extract and fractions from *A. cepa* in STZ-induced diabetic rats. The raw extract and the butanol fraction were proved to be effective within a dose level of 200 mg/kg. The ethanolic extract, particularly, was the most effective in reducing serum TG, TC and LDL within insulin group. The treatment with ethanolic extract for 21 days induced 50.28, 72.78 and 64.55% reduction of plasma LDL, TG and total cholesterol and it was also able to increase HDL level in relation to diabetic control.

The same experiments were carried out by Ojieh and co-workers on the juice of *A. cepa* bulbs [89]. Onions were purchased from a local market in Nigeria, cut into small pieces, mashed and squeezed out. The antilipidemic activity of *A. cepa* was verified in STZ-induced diabetic male Wistar rats. It was demonstrated that *A. cepa* significantly decreased the total cholesterol level in a dose dependent manner.

Interestingly, all the onion by-products were investigated for their potential effects on serum lipid levels [90]. “Paste” (triturated onions), “juice” (the aqueous fraction), “bagasse” (the solid residue) and “purée” (obtained by passing whole onions for a sifter with mesh) were obtained from two *A. cepa* cultivars (‘Figueres’ and ‘Recas’) from Spain and were first analysed for their fibre composition and physicochemical properties. The “bagasse” was the by-product richest in fibre and its effects on serum lipid levels were assessed on rats fed with a cholesterol-rich diet. At the end of the experiments, serum TG and TC levels were significantly lower in rats fed with Bagasse supplementation than in the control group. Moreover, a highest level of serum HDL cholesterol (20%) was observed in animals fed with onion by-product supplementation.

The antihyperlipidemic activity of an *A. cepa* extract obtained by heating onions and then concentrating them at 92 °C after squeezing was also reported [82]. Serum triglyceride and FFA levels were measured in ZDF rats fed with a diet containing onion extract (3% and 5%, *w*/*w*) for 28 days. Serum TG and FFA levels were found to be significantly lower in the onion extract-fed groups than the control group. All these results demonstrate the antihyperlipidemic potential of *A. cepa* different extracts.

### 5.2. Diabetes

A number of studies in literature deals with the potential effectiveness of *A. cepa* in the treatment of diabetes. These works have been reviewed in 2014 by Akash and colleagues [102].

More recently, the antidiabetic potential of the ethanolic extract and fractions from *A. cepa* was verified in STZ–induced diabetic rats [58]. Samples induced a significant effect in blood glucose level in an acute antidiabetic study. The raw extract, particularly, caused a 66.0% decrease at 200 mg/kg per os after 24 h. Moreover, the potential pancreatic β-cells regeneration effect of *A. cepa* extract and fractions was assessed and a pancreatic regeneration in the form of nesidioblastosis was observed in treated rats.

The hypoglycaemic potential of the juice of *A. cepa* bulbs was instead verified in STZ-induced diabetic male Wistar rats by Ojieh and co-workers [89]. The normoglycemic and diabetic groups were treated with graded doses of the sample (0.4 g/100 gbw and 0.6 g/100 gbw). The administration of 0.4 g/100 gbw of *A. cepa* reduced by 50% the fasting blood glucose levels of diabetic rats.

Henagan and co-workers assessed the beneficial effects of red onions extract on C57BL/6J mice [91]. Onions were peeled, chopped and extracted with 80% ethanol. Alcohol was then removed through evaporation and the remaining liquid was lyophilized. Authors aimed to verify if red onion extract dietary supplementation would have the same effects on insulin sensitivity than quercetin alone. They also verified if the effects of *A. cepa* extract were linked to an upregulation of energy expenditure through a mechanism involving skeletal muscle mitochondrial function. Both quercetin and onion supplementation were able to decrease high fat diet-induced fat mass accumulation and insulin resistance and to increase energy expenditure. These beneficial effects could be related to an improved skeletal muscle mitochondrial number and function but they occur through differential regulation of mtDNA-encoded gene expression.

Campos and co-workers evaluated the hypoglycaemic potential of onion infusion, obtained by extracting with water onion bulbs from Brazil. Sample was demonstrated to be effective in alleviating the hyperglycaemia in STZ-induced diabetic rats [57].

### 5.3. Hypertension

The potential beneficial effects of *A. cepa* were assessed by Brull and co-workers in overweight-to-obese patients with hypertension [92]. Onion skins were extracted with ethanol. The quercetin content of the onion skin extract was determined by HPLC and capsules containing 54 mg of quercetin were produced. In this study, subjects received three capsules per day in a double-blinded, randomized, placebo controlled cross-over trial. Office BP and ABP were measured before and after the intervention. Overall, quercetin-rich onion did not affect 24 h ABP parameters and office BP in the total group. On the contrary, blood pressure was significantly affected in the subgroup of hypertensive patients: quercetin decreased 24 h systolic BP when compared with placebo, even if the mechanisms responsible for this BP-lowering was not elucidated.

### 5.4. Cardiovascular Diseases

Obesity, especially the central or visceral type, is a predisposing factor not only for the development of diabetes and hypertension but also cardiovascular diseases [103]. This related comorbidity is linked to the release of several induced inflammatory markers that might contribute to the cardiovascular outcome in obese people. As a matter of fact, the adipose tissue releases a large number of cytokines and bioactive mediators, such as leptin, adiponectin, TNF-α and IL-6, that are able to influence not only insulin resistance, diabetes and lipid levels, but also coagulation, fibrinolysis, inflammation and atherosclerosis. In obese patients, a variety of morphological adaptations in cardiac structure and hemodynamic function may be observed [104].

Onions have a long history of use in the treatment of cardiovascular disorders in the traditional medicine of different cultures and a number of studies deals with the potential benefits of its consumption, so that they were reviewed already in 1987 by Kender [105].

Choi and co-workers evaluated the potential beneficial effects of *A. cepa* peel extract on endothelial function in healthy overweight and obese patients [93]. Onions utilized in this study were purchased from Korea and were extracted with 60% aqueous ethanol solution at 50 °C. Obtained solution was then concentrated and processed with a freeze dryer to obtain a powder containing quercetin 100 mg/g. In this randomized double-blind, placebo-controlled trial, individuals received a capsule of onion peel extract containing 50 mg quercetin twice daily or placebo capsules for 12 weeks. During the study period, BP, heart rate and lipid profile were not significantly affected, while body weight and body mass index (BMI) were significantly reduced in the onion extract consumption group. These results were different from those observed by Brull and co-workers [92]. FMD and circulating EPCs were verified in order to evaluate endothelial function. In the onion treated group a significantly improvement in FDM values between baseline and 12-weeks follow-up was observed, while no differences were detected in the control group. The percentages of EPCs were significantly increased in the onion consumption group as well [93].

Interestingly, Park and co-workers [94] investigated the potential effect of *A. cepa* bulbs extract on ischemic heart injury. For the preparation of the extract, the outer skins of fresh onions were removed and onion bulbs were extracted with 70% methanol. *A. cepa* showed a preventive effect both in vitro, on ischemia/hypoxia-induced apoptotic death in heart-derived H9c2 cells and in vivo, on rat myocardial infarction model. The extract (10 g/Kg) was able to significantly reduce the infarct size and the apoptotic cell death.

## 6. *Allium cepa* L. Bioactive Compounds with Anti-Obesity Properties

**Figure 1:**
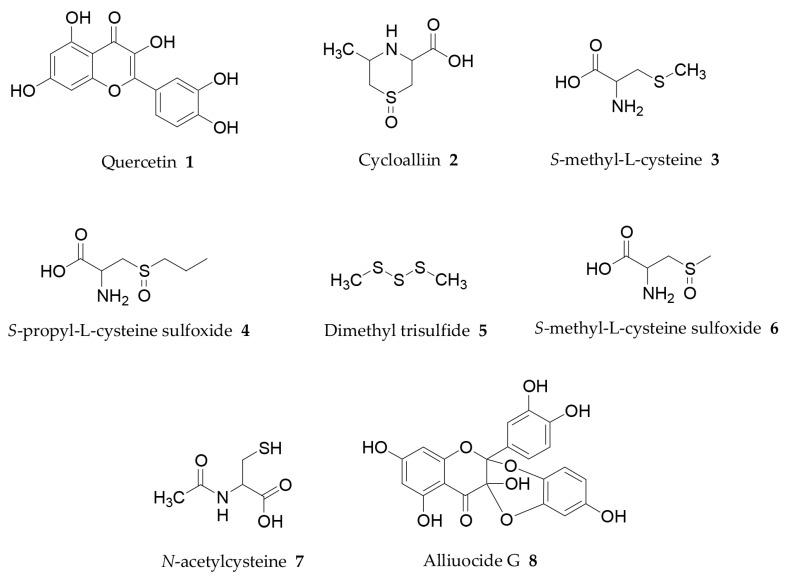
Structure of *A. cepa* chemical constituents with potential anti-obesity properties [82,84,85,88,106,107,108].

The structures of the most interesting onion phytochemical compounds with regard to the beneficial effects in the treatment of obesity are shown in Figure 1.

### 6.1. Quercetin

Quercetin (**1**, Figure 1) and its glycosides are the most abundant onion flavonoids [44]. Quercetin usually occurs in onion as 4′-monoglucoside and 3,4′-diglucoside. These molecules have a much greater bioavailability than quercetin [88].

The antihyperlipidemic effects of quercetin supplementation were verified by Lee and co-workers but it was reported that the intake of this flavonoid was less effective than onion powder intake [88]. The total cholesterol concentration slightly decreased in the liver of treated rats in the quercetin group but was not significantly different from the group fed with a high fat diet (82.4 ± 5.3 and 90.3 ± 3.9 mg/g wet wt, respectively).

Moreover, this flavonols was demonstrated to be an inhibitor of adipogenesis [84]. Quercetin treatment significantly decreased lipid accumulation in 3T3-L1 preadipocyte cells.

Moreover, Kaur [106] tested the anti-obesity potential of quercetin isolated from *A. cepa* in combination to curcumin and piperine, from *Curcuma longa* L. and *Piper nigrum* L., respectively. Combinatorial preparation was realized by suspending curcumin: piperine: quercetin (94:1:5) in 5% Gum Acacia and 0.5% tween 80 and it was administered per os at doses of 500, 1000 and 2000 mg/kg of body weight to high fat diet and low-dose streptozotocin-induced rats. After 28 days of treatment, plasma glucose level, triglyceride, LDL and cholesterol levels were significantly reduced (68.84%, 88.94%, 26.38% and 50.23%, respectively). Moreover, an improved glucose tolerance was observed.

Quercetin seems to play a potential role also in diet-induced thermogenesis, as it is also responsible for the browning effect of onion peel observed by Lee and co-workers, who reported that onion is able to modify the characteristics of white adipocytes to those of brown-like adipocytes in 3T3-L1 fibroblasts [85]. The quercetin-associated browning effect seems to be mediated in part by the activation of AMP-activated protein kinase.

### 6.2. Sulphur-Containing Components

*A. cepa* is known to contain a variety of sulphur-containing compound, also responsible for the characteristic pungent taste and lachrymatory factor [109].

The inhibitory effects of sulphur-containing compounds found in fresh and/or cooked onion on the differentiation of white adipose cells has been recently reported [82]. A strong inhibition of adipogenesis was demonstrated for cycloalliin (**2**), *S*-methyl-L-cysteine (**3**), *S*-propyl-L-cysteine sulfoxide (**4**), dimethyl trisulfide (**5**) and *S*-methyl-L-cysteine sulfoxide (**6**). According to these results, the anti-obesity effect of the onion extract could be in part related to these compounds.

Souza and co-workers investigated the potential beneficial effects of another organosulfur from *Allium* species: *N*-acetylcysteine (**7**) [107]. The influence on high-sucrose diet-induced obesity, lipid profile and in vivo LDL oxidation and serum oxidative stress was assessed on male Wistar rats. Obtained results demonstrated that this compound was able to improve the high-sucrose diet induced obesity.

### 6.3. Flavonoid Alliuocide G

A new flavonoid named alliuocide G (**8**) was isolated by Mohamed from the ethyl acetate fraction of *A. cepa* [108]. This molecule showed in vitro α-amylase inhibitory activity and radical scavenging potency.

## 7. Negative Results

Most of the studies focusing on *A. cepa* biological activity confirm the anti-obesity potential of this species. Conversely, a lack of effectiveness has also been reported.

Brull and co-workers [92] tested the beneficial effects of *A. cepa* in overweight-to-obese patients with hypertension and reported that quercetin-rich onion supplementation did not significantly affect body weight and did not influence serum total cholesterol, LDL-cholesterol, HDL-cholesterol neither in the total study group nor in the subgroup of hypertensive patients.

The same authors [110] recently also reported that quercetin from onion extract did not attenuate postprandial metabolic responses such as lipemia and insulinemia induced by a test meal rich in energy, fatty acids and carbohydrates, in overweight-to-obese hypertensive patients.

## 8. Conclusions

Onion is a plant with a long history of traditional medicinal uses. The extensive studies conducted in the last years confirmed that this species is a rich source of putative health-promoting phytochemicals, including flavonoids and organosulfur compounds. A number of works deal with the potential beneficial effects of onion dietary supplement. *A. cepa* extracts, its fractions and its identified bioactive components can induce their effects troughs different mechanisms of action: pancreatic lipase inhibition, adipogenesis inhibition and energy expenditure increase have been documented. Moreover, a substantial number of studies have proven the efficacy of *A. cepa* in the treatment of pathological conditions linked to obesity, such as hyperlipidaemia, diabetes, hypertension, cardiovascular diseases and inflammatory state. At the state of the art, quercetin and organosulfur compounds seems to be the compounds responsible for the anti-obesity potential of *A. cepa* and so the most promising molecules for a therapeutic application.

On the other hand, a very few number of studies contradicted these positive results. However, a comparative analysis of data cannot be easily performed. It has to be taken into account that the studies concerning *A. cepa* here reviewed were conducted on different plant materials, grown and harvested in different pedo-climatic conditions and whose active principles were extracted through more than one extraction techniques and solvents. Actually, for these reasons, the phytochemical composition of investigated samples might vary even consistently. These differences should be taken into account in further investigations on the potential use of onion in effective formulations intended for weight control and /or the treatment of obesity related comorbidities.

Edible plants have a great potential as functional ingredients able to induce anti-obesity effects. They represent an effective tool in the fight against overweight and obesity. Onion enriched food could be also taken into account for their potential use in obesity treatment and prevention. In the future research, both genetic engineering, for the improvement of the active metabolites synthesis and food industry and its innovative approaches, might play an important role in the development of healthier foods useful against obesity.

## Figures and Tables

**Table 1:** Beneficial effects of A. cepa on obesity and related comorbidities.

Effect	Extract	Test Subjects	Ref.
Pancreatic lipase inhibition	Peel extract obtained with fermented alcohol	-	[79]
	Leaves hydroalcoholic extract	-	[80]
	Onion juice	-	[81]
Adipogenesis inhibition	Heated fresh onion at 92 °C	Rat white preadipocyte cells	[82]
	Quercetin-rich onion peel 60% aqueous ethanol extract	3T3-L1 preadipocyte cells	[83]
	Quercetin-rich onion peel hydroalcoholic extract	3T3-L1 preadipocyte cells	[84]
Energy expenditure increase	Onion peels 60% aqueous ethanol extract	3T3-L1 preadipocyte cells	[85]
Influence on expression of inflammatory mediators from the adipose tissue	Onion peel hydroalcoholic extract	Dawley rats	[86]
Antihyperlipidemic activity	Petroleum extract	Rats	[87]
	Peel extract	Mice	[79]
	Onion powder	Male SD rats	[88]
	Ethanolic extract and fractions	STZ-induced diabetic rats	[58]
	Bulbs juice	STZ-induced diabetic male Wistar rats	[89]
	Different onion by-products	Rats	[90]
	Heated fresh onion at 92 °C	ZDF rats	[82]
Hypoglycaemic potential	Ethanolic extract and fractions	STZ-induced diabetic rats	[58]
	Bulbs juice	STZ-induced diabetic male Wistar rats	[89]
	Peeled onions extracted with 80% ethanol	C57BL/6J mice	[91]
	Bulbs infusion	STZ-induced diabetic rats	[57]
Blood pressure lowering effect	Onion skins ethanolic extract	Overweight-to-obese patients with hypertension	[92]
Benefits on endothelial function	Hydroalcoholic extract obtained at 50 °C	Healthy overweight and obese patients	[93]
Preventive effect on ischemic heart injury	Onion peeled bulbs hydroalcoholic extract	Heart-derived H9c2 cells and rats	[94]

## Abbreviations

The following abbreviations are used in the manuscript:

3T3-L1	mouse fibroblasts
ABP	ambulatory blood pressure
A1235A	human glioblastoma cell line
ABTS	2,2′-azino-bis(3-ethylbenzothiazoline-6-sulphonic acid)
B16F10	murine melanoma cells
BMI	body mass index
BP	blood pressure
C/EBPα	CCAAT/enhancer-binding protein α
Caco-2	human colorectal adenocarcinoma cells
CAT	catalase
DPPH	2,2-diphenyl-1-picrylhydrazyl radical
EPCs	endothelial progenitor cells
ER	extended release
FAO	Food and Agriculture Organization of the United Nations
FAS	fatty acid synthase
FFA	free fatty acids
FMD	flow-mediated dilation
FST	forced swimming test
Gbw	gram body weight
GPDH	glycerol 3-phosphate dehydrogenase
GSH	glutathione peroxidase
H9c2	cardiac muscle cell line
HDL	high density lipoprotein
HepG2	human hepatocellular carcinoma cells
HF	high fat diet
IL-6	interleukin-6
LDL	low-density lipoproteins
LPO	lipid peroxidase
MDA-MB-231	human breast cancer
NO	nitric oxide
ORAC	oxygen radical absorbance capacity
PPAR-γ2	peroxisome proliferator-activated receptor γ2
SOD	superoxide dismutase
SR	sustained release
STZ	streptozotocin
TC	total cholesterol
TG	triacylglycerides
TNF-α	tumor necrosis factor-α
ZDF	Zucker diabetic fatty
